# Morphometric measurements and the relationship with body weight in the Sudanese Dorcas Gazelle and Bohor Reedbuck

**DOI:** 10.1038/s41598-022-20156-0

**Published:** 2022-10-07

**Authors:** Amr M. A. Rashad, Taha K. Taha, Ahmed E. Mahdy, Mahmoud A. Aziz, Ahmed E. Badran

**Affiliations:** 1 Animal and Fish Production Department, Faculty of Agriculture (Alshatby), Alexandria University, Alexandria, 11865 Egypt; 2 Wildlife Department, Faculty of Animal Production, Gezira University, Wad Madani, Sudan; 3 Present Address: Faculty of Agriculture, Alexandria University, El-Shatby, Alexandria, Egypt

**Keywords:** Evolution, Zoology

## Abstract

The objectives were to describe the morphometric measurements and determine the best model for estimating the relationship between body weight and morphometric measurements of the two Sudanese antelopes, Dorcas Gazelle (*Gazella dorcas*) and Bohor Reedbuck (*Redunca redunca*). Twenty-four animals belonging to two Sudanese antelope species, six males and six females from each species were used. Data on body weight and body measurements were recorded from each species. Averages of body weight and morphometric traits of Bohor Reedbuck were larger than the corresponding values of Dorcas Gazelle, while the opposite was true in the case of horn thickness, which was larger in the later. Stepwise regression analysis indicated that, the best model for Dorcas Gazelle had the variables neck length, belly girth and chest height, while Bohor Reedbuck had body length, head length, neck length, tail length, chest girth and pelvic height. These variables explained 82% of the total variation in body weight of Dorcas Gazelle, and 92% of the variation of Bohor Reedbuck. These results are discussed in relation to morphometric measurements reported for antelopes elsewhere.

## Introduction

The venison market is an emerging branch in the meat industry. Venison is not as commonly available as beef, although there is an increasing demand for it as an alternative source of red meat. In recent years, an interest in venison has grown^1^. Venison production has increased to be about two million tons annually^2^. Unlike the traditional livestock species, deer is skipped in the intensive breeding system typical of nowadays meat industry. However, in the developed countries deer farming is increasing in popularity.

Currently, there has been a growing interest in establishing deer farming for the purpose of meat production. Some well-known examples are the Red deer in New Zealand (*Cervus elaphus*)^3^, the Wapiti (*Cervus elaphus canadensis*) and other species in Canada^4^, the Reindeer (*Rangifer tarandus*) in Arctic areas^5^ and the Fallow deer (*Dama dama*) in Italy^6^.

Two antelope species, Dorcas Gazelle (*Gazella dorcas*) and Reedbuck (*Redunca redunca*) are found in the Sudan^7,8^. Although the distribution of both species is restricted, each may potentially contribute to domestic food supply and commerce. However these wild ungulates remain overlooked as a food source and no information exists on their production potential and meat quality in the country.

Previous studies have emphasized the importance of some non-genetic factors on body weight growth of different deer species. Sex and age are considered the main non-genetic factors affecting body weight growth characteristics and yields^9,10^. Data on the effect of sex and age on morphometric traits and body weight growth of Dorcas Gazelle and Bohor Reedbuck are not found in the literature^7^.

The objectives of this study are to:

1. Describe the morphometric measurements of Dorcas Gazelle and Bohor Reedbuck.
2. Determine the best model for estimating the relationship between body weight and the morphometric measurements of both species.

## Materials and methods

### Study area

This study was carried out in the Republic of Sudan, namely Al Sabaloka (Jebel AL-Hassaniya, game reserve located in the Nile River State about 82 km north of Khartoum), Dinder National Park (DNP, park located 470 km south east of Khartoum.). The Dorcas Gazelle is from Al Sabaloka protected area, while the Bohor Reedbuck is from the DNP.

### Data collection

The number of individuals included in this study was 24, including 12 from each species (6 males and 6 females). Each individual was randomly chosen. Dorcas Gazelles were live-captured from Al Sabaloka (Jebel Al-Hassaniya) during April 2018 to July 2018. Bohor Reedbucks were live-captured from the DNP during December 2017 to March 2018. The age of animals was determined by dentition.

### Data collection

Body weight (BW) was measured using a hanging scale, to the nearest 0.5 kg. Immediately after weighing, the following morphometric measurements were recorded to the nearest cm, while the horn thickness measurements were taken in mm:

- Body length (BL), measured from the dorsal base of the head to the base of tail.
- Head length (HL), measured from the tip of the muzzle to in-between the horns.
- Neck length (NL), measured from behind the mandible to the first rib.
- Ear length (EL), measured from the base of the ear to its upper tip.
- Tail length (TL), measured from the base to the tip, excluding the terminal hair bristles.
- Horn length (HOL), measured from its base to the tip.
- Horn thickness (HT), measured at three regions, base, middle and apex.
- Chest girth (CG), measured as the circumference behind the wither and shoulders.
- Belly girth (BG), measured as the circumference at the middle of the belly.
- Chest height (CH), measured vertically from the chest to the ground.
- Pelvic height (RH), measured vertically from the rump to the ground.
- Hind leg length (HLG), measured vertically from the back down to hoof of the hind limb.
- Belly height (BG), measured from the belly down to the ground.
- Hock joint height (HJH), measured from the hock joint to the ground.

**Figure 1:**
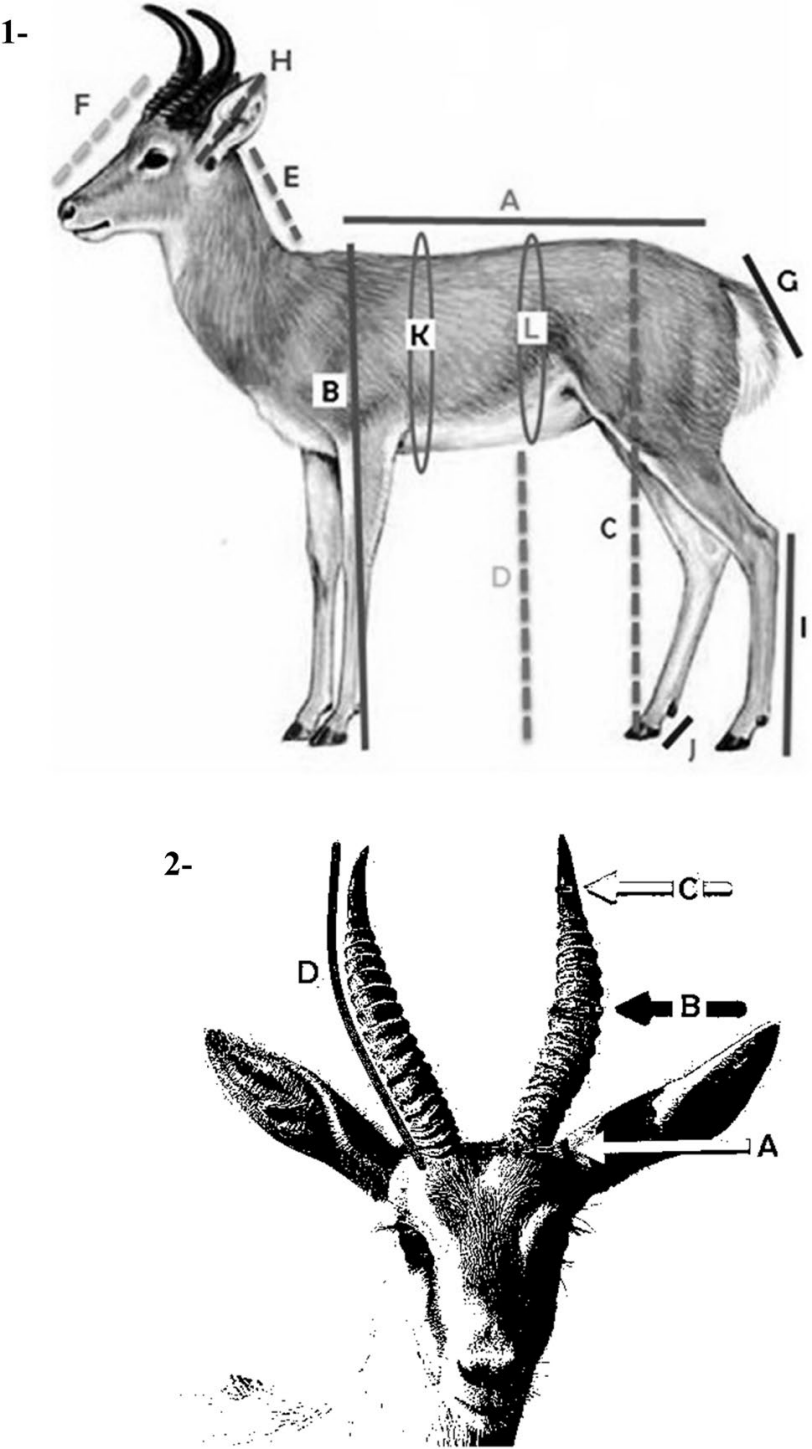
Animal body and horn measurements. 1. Body length (A), Head length (F), Neck length (E), Ear length (H) Tail length (G), Chest girth (K) Belly girth (L), Chest height (B), Pelvic height (C), Hind leg length (I), Belly height (D)and Hock joint height (J). 2. Horn length (D), Horn thickness (A, B and C).

The height measurements were taken using a graduated measuring stick. The length and circumference measurements were measured with a tape ruler, while the thickness was measured by Vernier caliper. All measurements were carried out by the same person in order to avoid between-individual variations. Figure 1 illustrates the abovementioned measurements.

### Statistical analysis

Descriptive statistics were calculated using the SUMMARY procedure of SAS^11^. To estimate collinearity between morphometric measurements, the following steps were undertaken.

$$ {\text{VIF}} = \frac{1}{{1 - {\text{r}}_{12}^{2} }} $$

Collinearity leads to large variances for estimated coefficients between variables^12^. The Variance Inflation Factor (VIF) represents the increase in variation due to the high correlation between variables (i.e., collinearity). No absolute standard for judging the magnitude of the VIF exists, but there is indication of collinearity when VIF > 10.00, according to Gill^14^. All VIF values are equal to 1.00 for uncorrelated variables. A further step for testing collinearity is to calculate the tolerance value (T)

$$ {\text{T}} = \frac{1}{{{\text{r}}^{{{\text{ii}}}} }} $$

where r^ii^ is the diagonal element of the inverse of the matrix^14^. If T ≤ 0.10 for any x-variable, then it should be excluded from further analysis.

Deleting unwanted variables from a given model avoids apparent collinearity and improves the efficacy of the analysis. According to Weisberg^12^, variables with small $\frac{{\left| {{\text{B}}_{{\text{j}}} } \right|}}{{\upsigma }}$ would be desirable, where B_j_ is the regression coefficient of x_j_ variable and σ is the square root of residual mean square. Analysis was performed on the full model in order to obtain this quantity. The full model was defined as:

$$ {\text{y}} = {\text{B}}_{0} + \left[ {{\text{B}}_{{1}} {\text{x}}_{{\text{i}}} } \right] $$

Stepwise regression following backward elimination was used to select the best models to estimate variation in body weight. Criteria used to select the best model among the candidate set were:

- R^2^ values to quantify the proportion of variability explained by a given model. A limitation of this criterion is that all subsets compared must have the same number of parameters.
- Adjusted R^2^ (adj. R^2^).
  $$ {\text{Adj}}.{\text{ R}}^{2} = 1 - \left[ {\frac{{\left( {{\text{n}} - 1} \right)}}{{\left( {{\text{n}} - {\text{p}}} \right)}}} \right]\left( {1 - {\text{R}}^{2} } \right) $$

where n is the number of observations, and p is the number of variables in the model.

c. The C_p_ statistic which is defined by Weisberg^12^ as
  $$ {\text{C}}_{{\text{p}}} = \left( {\frac{{{\text{RSS}}}}{{{\upsigma }^{2} }}} \right) - \left( {{\text{n}} - 2{\text{p}}} \right) $$
  where RSS is residual sum squares from a p variate subset model, where σ^2^ is the residual mean square from the full model, and n and p are explained above. The C_p_ is used to assess the ‘goodness of fit’ of a regression model. It is applied in the context of model selection, where a number of variables are available for predicting some outcome, and the goal is to find the best model involving a subset of these predictors. A small value of C_p_ means that the model is relatively precise. The C_p_ criterion is more powerful than R^2^ and adjusted R^2^, because it is a function of n, p, ϭ^2^ and RSS.

These statistics were calculated using the regression (PROC REG) and the correlation (PROC CORR) procedures of SAS^11^.

### Approval for animal experiments

All animals and experimental procedures in this study were supervised and approved by the Institutional Animal Care and Use Committee of Alexandria University, Egypt. Also, all procedures and experimental protocols were in accordance with the ethical standards as laid down in the 1964 Declaration of Helsinki and its later amendments.

## Results and discussion

### Body weight and morphometric measurements

Table 1 summarizes the means, ranges and standard deviations of body weight and morphometric measurements of Dorcas Gazelle and Bohor Reedbuck. The mean body weight of Bohor Reedbucks was larger than the corresponding value of Dorcas Gazelles (46.54 and 12.25 kg, respectively). Similarly, the other morphometric measurements of Bohor Reedbucks were larger than those of the Dorcas Gazelles, except the outer thickness of horns (base, mid, apex) which were larger in Dorcas Gazelles (19.43, 15.9 and 8.05 mm, respectively) than those of the male Bohor Reedbucks (3.67, 2.63 and 0.52 mm, respectively). The range in body weight of Bohor Reedbucks (18–65 kg) is within that reported by Abdel Hameed^15^ for Sudanese Reedbuck (36–80 kg). Nowak^16^ reported mean head and body lengths equal to 110 to 160 cm, which are considerably larger than the findings in this study for both traits in Bohor Reedbucks. The results in the literature for the horn length ranged between 20–41 cm, and the tail length between 15–44 cm^15–17^. Ahmed^7^ reported body length ranging between 104–114 cm for males and 80–110 cm in females. The horn length range was 28–35 cm, the girth range was 25–35 cm in males and 24–27 cm in females. The ear length range was 13–17.5 cm in the males and 13–15 cm in females. The body weight range was 37–58 kg in males, and 19–35 kg in females.

**Table 1:** Means (± SD) for body weight and some body measurements of Sudanese Dorcas Gazelle and Bohor Reedbuck antelopes.

Trait	Dorcas Gazelle	Bohor Reedbuck
Body weight (kg)	12.25 ± 1.62	46.54 ± 15.09
Body length (cm)	50.17 ± 3.66	77.00 ± 6.70
Head length (cm)	15.25 ± 2.56	18.96 ± 2.12
Neck length (cm)	24.17 ± 4.49	30.67 ± 5.93
Ear length (cm)	14.96 ± 1.05	15.42 ± 0.79
Tail length (cm)	14.75 ± 1.86	19.00 ± 1.95
Horn length (cm)*	17.42 ± 3.60	27.50 ± 14.39
Chest girth (cm)	52.21 ± 4.60	82.75 ± 12.63
Belly girth (cm)	52.92 ± 5.11	93.00 ± 14.54
Chest height (cm)	56.17 ± 3.76	83.00 ± 6.89
Pelvic height (cm)	62.33 ± 2.77	88.08 ± 4.64
Belly depth (cm)	10.25 ± 5.64	24.08 ± 7.30
Hind leg length (cm)	27.17 ± 2.17	36.83 ± 1.70
**Horn thickness (mm)**		
Base-diameter	19.43 ± 5.78	3.67 ± 1.33
Mid-diameter	15.97 ± 4.60	2.63 ± 1.16
Apex-diameter	8.05 ± 4.98	0.52 ± 0.25
Belly height (cm)	45.92 ± 6.33	58.92 ± 4.74
Hock joint height (cm)	4.88 ± 1.35	7.62 ± 0.76

*Averages of horn length and thickness of reedbuck were calculated from males only, as there are no horns present in the females.

*SD* Standard deviation.

Brouin^18^ reported a mean value of 20 kg for body weight of Dorcas Gazelles in Niger. Similarly, Oboussier^19^ noted that the mean body weight of Dorcas Gazelles in Chad was 19 kg. Yom-Tov et al.^20^ reported mean body weight was 16 kg, ranging between 14.6–18.2 kg. These authors also reported mean head and body length of Dorcas Gazelles from Sinai and the Sudan was 95.5 cm, ranging between 89.0–101.4 cm in males, and 95.2 cm, ranging between 88.5–101.0 cm in females. They stated that the tail length of Dorcas Gazelles was about 11–16% of the lengths of head and body in Sinai, 17.5–17.7% in Niger, and 21.2–21.5% in Chad. These results differ from the findings of our study where we obtained lower values of head and body lengths, with 15.25 cm for head length and 50.17 for body length. Yom-Tov et al.^20^ stated that the ear length was longer in the Sahara (14.8–17.7% of the head and body length, compared to 14.0–15.8% in Sinai). The variation among populations may be attributed to the different geographic areas and environmental circumstances under which the Dorcas Gazelles live^20^.

Groves^21^ stated that the mean horn length of females Dorcas Gazelles was about 62% of that of males in Somalia, but nearly 80% in the Sahara. Yom-Tov et al.^20^ reported that the horns lengths of Dorcas Gazelles vary from 20.1–26.6 cm depending on the geographical region in which the animal is living, which are lower than those measured in the present study. Wura^22^ stated that the Dorcas Gazelle is one of the smallest in size of all antelopes. The height at shoulder is only 53–76 cm, body length 90–110 cm. tail length 15–20 cm and body weight 15–20 kg.

### Best model estimating the relationship between body weight and morphometric measurements

Measurements of the animal's relative body shape dimensions can be considered as indirect indicators for the degree of its meat leanness^23^. Many attempts have been made to estimate body weight from of morphometric measurements in different livestock species^24–28^. By contrast, research work on estimation of body weight from morphometric measurements in deer species is very scarce^29–31^. Herein, we estimated the relation between body weight and several morphometric measurements of Dorcas Gazelle and Bohor Reedbuck, utilizing body length [x_1_], head length [x_2_], neck length [x_3_], ear length [x_4_], tail length [x_5_], chest girth [x_6_], belly girth [x_7_], chest height [x_8_], pelvic height [x_9_] and belly depth [x_10_].

Correlation coefficients among the body measurements for Dorcas Gazelles and Bohor Reedbucks are presented in Table 2. The results show that, correlation coefficients among morphometric measurements of Dorcas Gazelles ranged between − 0.64 (between belly depth and neck length) and 0.79 (between body length and chest girth).

**Table 2:** Correlation coefficients between body measurements (cm) of Dorcas gazelle and Bohor reedbuck.

Traits	Head length	Neck length	Ear length	Tail length	Chest girth	Belly girth	Chest height	Pelvic height	Belly depth
**Dorcas gazelle**									
Body length	0.69*	0.69*	0.55	0.06	0.79**	− 0.30	0.49	0.56*	− 0.54
Head length		0.56*	0.71**	0.05	0.59*	− 0.39	0.45	0.19	− 0.21
Neck length			0.40	0.11	0.70*	− 0.50	0.24	0.13	− 0.64*
Ear length				0.29	0.69**	− 0.09	0.48	0.52	− 0.17
Tail length					0.35	− 0.47	0.19	0.58*	− 0.08
Chest girth						− 0.35	0.60*	0.53	− 0.22
Belly girth							− 0.52	− 0.13	0.27
Chest height								0.43	0.14
Pelvic height									− 0.26
**Bohor reedbuck**									
Body length	0.91**	0.96**	0.89**	0.87**	0.99**	0.98**	0.95**	0.77**	0.80**
Head length		0.84**	0.85**	0.97**	0.89**	0.88**	0.93**	0.85**	0.55
Neck length			0.94**	0.85**	0.98**	0.99**	0.93**	0.61*	0.90**
Ear length				0.88**	0.94**	0.94**	0.90**	0.56	0.81**
Tail length					0.87**	0.86**	0.88**	0.74**	0.55
Chest girth						1.00**	0.96**	0.72**	0.85**
Belly girth							0.95**	0.69*	0.87**
Chest height								0.78**	0.78**
Pelvic height									0.34

*Significant at *P* < 0.05.

**Significant at *P* < 0.01.

By contrast, the corresponding correlation coefficients in the case of Bohor Reedbucks ranged between 0.34 (between belly depth and pelvic height) and unity (between belly girth and chest girth), Table 2. Correlation coefficients among the morphometric measurements of Dorcas Gazelles were all lower than 0.80, indicating that there may be no collinearity problem among these variables. The collinearity problem among morphometric measurements of Bohor Reedbuck were greater, as the majority of the correlation coefficients were larger than 0.80. However, the high correlation coefficients are not necessarily indicative of collinearity so the VIF was employed. The VIF for the morphometric measurements of Dorcas Gazelles and Bohor Reedbucks are presented in Tables 3 and 4. The VIF values for the relationship between morphometric measurements of Dorcas Gazelle were lower than 10, indicating that there was no collinearity problem among these variables. Several VIF values for the relationship between morphometric measurements of Bohor Reedbuck were > 10, indicating a collinearity problem. The VIF values between body length and each of neck length, chest girth, belly girth, and chest height, between head length and tail length, between neck length and each of chest girth and belly girth, between chest girth and each of belly girth and chest height, and between belly girth and chest height were > 10.

**Table 3:** Variance inflation factors (VIF) for the relationships between body dimensions (cm) of Dorcas gazelle.

Traits	Head length	Neck length	Ear length	Tail length	Chest girth	Belly girth	Chest height	Pelvic height	Belly depth
Body length	5.85	11.73	4.79	4.05	33.70	27.77	10.49	2.45	2.78
Head length		3.32	3.63	15.71	5.00	4.42	7.05	3.55	1.43
Neck length			8.74	3.55	32.95	39.90	7.01	1.58	5.12
Ear length				4.44	8.24	8.40	5.20	1.45	2.90
Tail length					4.21	3.95	4.36	2.22	1.44
Chest girth						124.32	14.35	2.06	3.69
Belly girth							10.76	1.90	4.01
Chest height								2.52	2.54
Pelvic height									1.13

**Table 4:** Variance inflation factors (VIF) for the relationships between body dimensions (cm) of Bohor reedbuck.

Traits	Head length	Neck length	Ear length	Tail length	Chest girth	Belly girth	Chest height	Pelvic height	Belly depth
Body length	1.91	1.90	1.45	1.00	2.67	1.10	1.31	1.45	1.41
Head length		1.47	2.02	1.00	1.54	1.18	1.25	1.04	1.04
Neck length			1.19	1.01	1.94	1.33	1.06	1.02	1.70
Ear length				1.10	1.89	1.01	1.30	1.37	1.03
Tail length					1.14	1.28	1.04	1.51	1.01
Chest girth						1.14	1.58	1.39	1.05
Belly girth							1.38	1.02	1.08
Chest height								1.23	1.02
Pelvic height									1.07

The T values and the diagonal elements of the inverse of the correlation matrix are presented in Table 5. High T values were observed for all morphometric measurements of both species. Thus, the majority of these variables exhibit collinearity and should be removed from the model.

**Table 5:** Diagonal elements (r^ii^) of the inverse of the correlation matrix and tolerance values (T) for body measurements of Dorcas gazelle and Bohor reedbucks.

Trait	Dorcas Gazelle		Bohor reedbuck	
	T	r^ii^	T	r^ii^
Body length (cm)	0.003	303.030	0.003	342.466
Head length (cm)	0.006	172.712	0.001	869.565
Neck length (cm)	0.048	20.695	0.003	383.142
Ear length (cm)	0.007	145.349	0.018	57.110
Tail length (cm)	0.047	21.336	0.005	183.150
Chest girth (cm)	0.007	134.409	0.001	826.446
Belly girth (cm)	0.012	85.543	0.004	225.734
Chest height (cm)	0.018	54.318	0.006	167.785
Pelvic height (cm)	0.040	25.088	0.023	42.626
Belly depth (cm)	0.009	106.496	0.006	163.399

### Model selection

The $\frac{{\left| {{\text{B}}_{{\text{j}}} } \right|}}{{\upsigma }}$ values are presented in Table 6 for all morphometric measurements included in the full model for both species. The smallest values in the case of Dorcas Gazelles were observed for ear length, body length, tail length and belly depth, accounting for − 0.87, − 0.46, − 0.18 and − 0.05, while the corresponding values obtained for Bohor Reedbucks were − 2.24, − 1.18, − 1.05, − 0.56, − 0.38 and − 0.09, representing head length, ear length, neck length, pelvic height, chest height and belly girth, respectively. Some of these variables must be removed from the model. This conclusion was confirmed by performing stepwise regression following backward elimination. The candidate models and their respective R^2^, adj. R^2^, C_p_ and number of parameters (p) for Dorcas gazelle and Bohor reedbucks are presented in Tables 7 and 8. Based on the R^2^ values, the best two models in the case of Dorcas Gazelles were the full model (model one) and that with belly depth excluded (model two). However, model four was the best model based on the adjusted value of R^2^ and model eight was the best model based on C_p_ criterion. It should be noted that model eight had the smallest number of predictors (e.g. neck length, belly girth and chest height). These three variables explained 82% of the total variation in body weight of Dorcas gazelle. The full model explained about 96% of the total variation in body weight, which means that the remaining seven variables explained about 14% of the total variation. Surprisingly, the adjusted R^2^ value of model number eight is similar to the corresponding value of the full model. This suggests that model number eight is the best for estimating body weight of Dorcas Gazelles.

**Table 6:** The quantity $\frac{{\left| {{\text{B}}_{{\text{j}}} } \right|}}{{\upsigma }}$ obtained from the full model for Dorcas gazelle and Bohor reedbucks.

Traits	Dorcas Gazelle	Bohor reedbucks
Body length (cm)	− 0.46	1.01
Head length (cm)	0.56	− 2.24
Neck length (cm)	0.23	− 1.05
Ear length (cm)	− 0.87	− 1.18
Tail length (cm)	− 0.18	3.24
Chest girth (cm)	0.12	1.27
Belly girth (cm)	0.24	− 0.09
Chest height (cm)	0.30	− 0.38
Pelvic height (cm)	0.44	− 0.56
Belly depth (cm)	− 0.05	0.28

**Table 7:** Coefficients of determination (R^2^), Adjusted (Adj) R^2^, C_p_ and number of parameters (p) for the best models to predict body weight in Dorcas gazelle.

No	Model*										R^2^	Adj. R^2^	Cp	p
1	X_1_	X_2_	X_3_	X_4_	X_5_	X_6_	X_7_	X_8_	X_9_	X_10_	0.96	0.78	11.00	10
2	X_1_	X_2_	X_3_	X_4_	X_5_	X_6_	X_7_	X_8_	X_9_		0.96	0.85	9.01	9
3	X_1_	X_2_	X_3_	X_4_	X_5_		X_7_	X_8_	X_9_		0.95	0.86	7.08	8
4	X_1_	X_2_	X_3_	X_4_			X_7_	X_8_	X_9_		0.95	0.89	5.11	7
5	X_1_	X_2_	X_3_				X_7_	X_8_	X_9_		0.92	0.85	3.96	6
6		X_2_	X_3_				X_7_	X_8_	X_9_		0.89	0.83	2.46	5
7			X_3_				X_7_	X_8_	X_9_		0.86	0.81	1.31	4
8			X_3_				X_7_	X_8_			0.82	0.78	0.14	3

*Body length [x_1_], head length [x_2_], neck length [x_3_], ear length [x_4_], tail length [x_5_], chest girth [x_6_], belly girth [x_7_], chest height [x_8_], pelvic height [x_9_] and belly depth [x_10_].

**Table 8:** Coefficients of determination (R^2^), Adjusted (Adj) R^2^, Cp and number of parameters (p) for the best models to predict body weight in Bohor Reedbuck.

No	Model										R^2^	Adj. R^2^	Cp	p
1	X_1_	X_2_	X_3_	X_4_	X_5_	X_6_	X_7_	X_8_	X_9_	X_10_	1.00	1.00	11.00	10
2	X_1_	X_2_	X_3_	X_4_	X_5_	X_6_		X_8_	X_9_	X_10_	1.00	1.00	9.09	9
3	X_1_	X_2_	X_3_	X_4_	X_5_	X_6_		X_8_	X_9_		1.00	1.00	7.38	8
4	X_1_	X_2_	X_3_	X_4_	X_5_	X_6_			X_9_		1.00	1.00	5.59	7
5	X_1_	X_2_	X_3_		X_5_	X_6_			X_9_		0.92	0.85	3.82	6

Body length [x_1_], head length [x_2_], neck length [x_3_], ear length [x_4_], tail length [x_5_], chest girth [x_6_], belly girth [x_7_], chest height [x_8_], pelvic height [x_9_] and belly depth [x_10_].

The best models predicting body weight from body measurements in Bohor Reedbucks are presented in Table 8. Based on R^2^ and its adjusted version, the full model was as good as model number two, three and four. However, the fifth model should be the best model based on the C_p_ criterion. This model with six parameters explained 92% of the total variation in body weight of Bohor Reedbucks, while the remaining four explained about 8%. It is suggested that one may use this model for estimating body weight of Bohor reedbucks. Table 9 shows the regression coefficients of the suggested best models estimating body weight of Dorcas Gazelles and Bohor Reedbucks, based on the C_p_ criterion. Regression coefficients of the selected variables estimating body weight were all significant, both Dorcas Gazelles and Bohor Reedbucks. Attempts to estimate body weights from body measurements of cervids are generally scarce. Nieminen and Petersson^32^ estimated live weight in semi-domesticated reindeer *(Rangifer tarandus tarandus* L.). They reported significant linear regressions between live weight with back length and chest girth. Fruziński et al.^33^ reported a significant correlation between body weight with body length (r = 0.70), hind foot length (r = 0.51) and shoulder height (r = 0.33).

**Table 9:** Regression coefficients (± standard errors) of the best two models predicting body weight from body measurements of Dorcas gazelle and Bohor reedbucks.

Dorcas gazelle		Bohor reedbucks	
Model	Regression coefficient	Model	Regression coefficient
Intercept	− 25.17 ± 7.03**	Intercept	− 73.00 ± 8.83**
Neck length	0.23 ± 0.06**	Body length	1.55 ± 0.22**
Belly girth	0.21 ± 0.06**	Head length	− 5.20 ± 0.97**
Chest height	0.37 ± 0.07**	Neck length	− 1.29 ± 0.51*
		Tail length	4.57 ± 0.80**
		Chest girth	1.30 ± 0.26**
		Pelvic height	− 0.63 ± 0.12**

*Significant at *P* < 0.05.

**Significant at *P* > 0.01.

Bundy et al.^29^ used data for eleven morphological measurements of white-tailed deer (*Orlocoileus virginianus*) to estimate body weight. The authors found that measurements of chest circumference, depth, width, and total body length were the most useful variables for estimating whole body weights. Similarly, Bartareau^34^ found that white-tailed deer maintained a similar proportion of body weight to sex, age, age^2^, chest girth^2^, and body length predictor variables while differences between the observed and estimated weights of the best model applied to a validation dataset were not significant. Body weights and morphometric measurements of Sambar deer (*Cervus unicolor*) from three states in Malaysia were studied by Idris et al.^35^. The authors reported that regression of body height, body length and heart girth had highly significant (*P* < 0.001) effects on body weight. These findings partially agree with results of the present study. We found that regression coefficients of body weight on neck length, belly girth and chest height in Dorcas Gazelles and on body length, head length, neck length, tail length, chest girth and pelvic height in Bohor Reedbucks were significant, and were the best models to describe body weights of Dorcas Gazelles and Bohor Reedbucks, respectively.

## Conclusion

The results obtained on the body weights and morphometric measurements of Dorcas Gazelles and Bohor Reedbucks encourage further studies utilizing a larger sample. We derived two models for estimating body weight from morphometric measurements, one for each species. However, validation of the obtained models with an independent dataset is necessary to evaluate the accuracy of our putative body weight-estimation models. Hypothetically, the models presented will enable accurate estimates of the body weight of individuals in cases where morphometric measurements are available, but the individuals were not weighed. These results provide a basis to formulate and parameterize body weight-estimation models for other antelope species and populations.

## Data Availability

The data that support the findings of this study are available from Faculty of Agriculture – Alexandria University but restrictions apply to the availability of these data, which were used under license for the current study, and so are not publicly available. Data are however available from the authors upon request and with permission of Faculty of Agriculture – Alexandria University.
